# The League of Mercy

**Published:** 1899-03-04

**Authors:** 


### The Institutional Workshop.

### THE LEAGUE OF MERCY.

### MEETING AT MARLBOROUGH HOUSE.

A large and influentially-attended meeting was held at Marlborough House on Wednesday, March 1st, to promote the establishment of a League of Mercy. H.R.H. the Prince of Wales occupied the chair.

For the purposes of the work of the League a president will be appointed for each Parliamentary division within the metropolis and the neighbouring counties, but it has only been possible in the time available before the departure of the Prince of Wales for the Continent to appoint presidents for 52 divisions out of a total of about 100 to which presidents will be elected to control the League organisation. The following have agreed to become presidents of the Parliamentary divisions given in brackets against their names, and most of them were present at the meeting: Duke of Westminster (St. George's, Hanover Square), Marquis of Lorne (Kensington, South), Marquis Camden (Tonbridge), Earl Carrington, G.C.M.G. (St. Pancras, West), Earl of Clarendon (Watford), Earl of Dartmouth (Lewisham), Earl of Mansfield (St. Pancras, North), Earl of March (Chichester), Earl of Onslow, G.C.M.G. (Westminster), Earl of Verulam (St. Albans), Lord Farquhar (Marylebone, West), Lord Harris, G.C.S.I., G.C.S.E. (Faversham), Lord James of Hereford (Chelsea), Lord Medway (Ashford), Lord Northbourne (St. Augustines), Lord Wolverton (Finsbury, Central), the Lord Mayor (City of London), Right Hon. Sir W. Hart-Dyke, Bart., M.P. (Dartford), Sir Whittaker Ellis, Bart. (Kingston), Right Hon. Sir Arthur Hayter, Bart. (East Berks), Sir Trevor Lawrence, Bart. (Reigate), Sir Fitzroy D. Maclean, Bart. (Hythe), Sir W. D. Pearson, Bart. (Colchester), Sir W. D. Crundall (Dover), Sir Donald Currie, K.C.M.G., M.P. (Paddington, North), Major-General Sir H. P. Ewart, K.C.B., K.C.V.O. (Maldon), Right Hon. Sir Charles Hall, K.C.M.G., M.P. (Finsbury, Holborn), Sir Henry Harben (Hampstead), Hon. W. F. D. Smith, M.P. (Strand), Mr. H. Cosmo O. Bonsor, M.P. (Epsom), Mr. Edmund Boulnois, M.P. (Marylebone, East), Mr. Sydney Buxton, M.P. (Poplar), Mr. Edward H. Byas (Bow and Bromley), Mr. E. Cassel (St. George's, Hanover Square), Mr. F. S. W. Cornwallis, M.P. (Maidstone), Mr. C. Gold, M.P. (Saffron Walden), General Goldsworthy, M.P. (Hammersmith), Mr. Frederick Gordon (Harrow), Mr. E. A. Hambro (Sevenoaks), Mr. John Harris (Whitechapel), Very Rev. S. R. Hole, D.D. (Rochester), Mr. Alexander Hubbard (Ealing), Mr. G. B. Hudson, M.P. (Hitchin), Colonel Hughes (Woolwich), Lieut.-Colonel Lockwood, M.P. (Epping), Mr. E. Macrory, Q.C. (Paddington, South), Hon. W. F. B. Massey-Mainwaring, M.P. (Finsbury, Central), Mr. P. A. Nairne (Camberwell), Mr. J. Round, M.P. (Harwich), Mr. J. Sebag-Montefiore (Isle of Thanet), Mr. H. C. Stephens, M.P. (Hornsey), Mr. A. H. Tarleton (Deptford), and Mr. W. P. Treloar (Croydon).

In opening the meeting, H.R.H. the Prince of Wales said: My Lords and Gentlemen,—I have invited you here this morning to discuss a very important question, and to ask for your assistance and co-operation in enabling me to carry it into effect. In my letter to the public, dated February 5th, 1897, when the Prince of Wales's Hospital Fund for London was instituted, I invited subscriptions of one shilling per annum and upwards from all classes to this Fund. I then said, "Our attention will be concentrated upon an endeavour to secure annual subscriptions from those who have not hitherto regularly contributed." It has been pointed out by the medical press, and represented to me personally, that, to accomplish this object successfully, some organisation is required to reach locally the classes to whom I specially referred. It is essential that any such organisation should be so devised as to prevent the possibility of competition with existing organisations for the benefit of hospitals like the Hospital Sunday and Hospital Saturday Funds, whilst it opens up new and avoids old ground by interesting those members of the community who have not hitherto subscribed regularly, if at all, to hospitals. About a year ago a committee was appointed, of which Mr. E. A. Hambro was chairman, which had under consideration many proposals and plans for devising some scheme which would meet the objects already explained. Infinite trouble was taken to test, as far as practicable, the various proposals submitted to the consideration of the committee, and to ascertain to what extent it would be feasible to interest a large number of new people in the work of the hospitals to the extent of giving a small annual subscription. In the end it was determined to organise a League of Mercy based upon the plan of the Guild founded by the late Duchess of Teck. The purpose for which the League of Mercy has been established is to promote the welfare and to further the objects of the Prince of Wales's Hospital Fund for London, and in every way, but especially by encouraging personal service on the part of large numbers of persons, to further the interests and to promote the adequate maintenance of hospitals and other institutions for the relief of sickness and suffering, and especially those institutions which are supported by voluntary contributions. To effectually carry out the work of the League London and the home counties will be divided into a number of districts representing the present Parliamentary divisions. A president will be appointed for each district, who will subdivide it into 30 smaller areas, to each of which he will appoint a vice-president. Each vice-president will select 20 members, each of whom will undertake to find 20 subscribers of one shilling a-year and upwards. In connection with the League an "Order of Mercy" has been established which will be conferred as a reward for gratuitous personal services only rendered in the relief of sickness, suffering, poverty, or distress, and which will have only one class. All persons recommended for admission to the Order must in the first instance be submitted by a president or a lady president, either on the recommendation of a vice-president or a lady vice-president, or otherwise to the Grand President. No person can be admitted to the Order unless his or her name has been approved and sanctioned by the Queen as Sovereign of the League on the submission of the Grand President. The Order may be worn on all occasions, but will not confer any rank, dignity, or social precedence. Provision has been made to effectually preserve pure this most honourable distinction, and no member of the League will be eligible to receive it who has not rendered the required gratuitous service to the League for five years at least. The organisation has the merit of simplicity. It is devised so as to prevent the multiplication of committees and complicated machinery, and has the further advantage of embodying a plan by which every penny of the money subscribed through the gratuitous personal service of its members shall go direct to the hospitals without the deduction of one penny for expenses. I have felt that in a work of this kind the ladies could render very material assistance, and it has therefore been decided to appoint a lady president in each Parliamentary division. I have the pleasure to inform you that the Princess of Wales has consented to become the Lady President—(hear, hear)—and that I myself desire to do all I can to help the good work by taking the office of President. (Hear, hear.) It is of course impossible to accurately forecast the result, but I am confident that, with the zealous co-operation of the presidents and lady presidents and of the ladies and gentlemen who will become vice-presidents and members, a very considerable sum must be added to the yearly income of the hospitals as the result of the work of the League of Mercy. In thanking all who have already consented to accept the office of president of the League in the various Parliamentary divisions, I may add that it is hoped that a large number of ladies and gentlemen will spontaneously offer their services as vice-presidents and members in each Metropolitan division as well as in the neighbouring counties. There is evidence to show that a great number of willing workers are available who have not previously had the opportunity to tender their gratuitous services and to devote a portion of their time on behalf of the hospitals. (Applause.)

His ROYAL HIGHNESS invited a discussion of the details of the scheme.

Lord CARRINGTON said he would like to ask if he was right in assuming from what he had heard that it was to be distinctly understood that the decoration was to be awarded for personal service only, and that no donation or subscription, however large, would entitle any person to so great an honour.

The PRINCE of WALES: I am very glad you have asked this question. What you say is quite correct, and I hope you will also observe that the Order is to be for one class only.

In the course of the discussion it was pointed out that the funds collected would, subject to such conditions as the Prince of Wales might decide upon, be paid over to the Prince of Wales's Hospital Fund.

Sir WILLIAM HART DYKE earnestly hoped that the fact that the money collected by the League was being raised for a great National Hospital Fund would be kept distinctly in view, and that it would always be distinctly understood that the first aim of the League was to collect for the Prince of Wales's Hospital Fund, and not for the local charities.

Colonel LOCKWOOD and Lord VERULAM supported what Sir W. Hart Dyke had said.

Sir HENRY BURDETT pointed out that the grounds for asking the home counties to give were, first, that a very large and increasing number of patients came to the London hospitals from those districts; and, secondly, that amongst the residents in those counties were a large number of most influential and wealthy people who had business relations with London, and who made their money in London. His Royal Highness had said that every penny which was subscribed to the League through the members would go into the coffers of the hospitals. This was so because, although at first the subscriptions of the presidents and vice-presidents would be devoted to expenses, ultimately if the League was a success, a capital sum would be available from a source which he need not then explain which would be sufficient to provide an income to pay all expenses.

The PRINCE of WALES nominated Sir Henry Burdett as treasurer, Dr. Collins and two other gentlemen as honorary secretaries, and Colonel W. Knollys as registrar.

A vote of thanks to the Prince of Wales for presiding was unanimously passed, and the meeting terminated.

---

